# TGFBR2-dependent alterations of exosomal cargo and functions in DNA mismatch repair-deficient HCT116 colorectal cancer cells

**DOI:** 10.1186/s12964-017-0169-y

**Published:** 2017-04-04

**Authors:** Fabia Fricke, Jennifer Lee, Malwina Michalak, Uwe Warnken, Ingrid Hausser, Meggy Suarez-Carmona, Niels Halama, Martina Schnölzer, Jürgen Kopitz, Johannes Gebert

**Affiliations:** 1 Department of Applied Tumor Biology, Institute of Pathology, University Hospital Heidelberg, Im Neuenheimer Feld 224, 69120 Heidelberg, Germany; 2 Department of Cancer Early Detection, German Cancer Research Centre (DKFZ), Im Neuenheimer Feld 224, 69120 Heidelberg, Germany; 3 Present address: Tissue Genesis, Suite 1000, Tissue Genesis Tower, 810 Richards Street, Honolulu, HI 96813 USA; 4 Functional Proteome Analysis and Core Facility Protein Analysis (B100), German Cancer Research Centre (DKFZ), Im Neuenheimer Feld 280, 69120 Heidelberg, Germany; 5 Department of General Pathology, Institute of Pathology, University Hospital Heidelberg, Im Neuenheimer Feld 224, 69120 Heidelberg, Germany; 6 Department of Medical Oncology, National Center for Tumor diseases (NCT), Tissue Imaging and Analysis Center, Bioquant, University Hospital Heidelberg, Im Neuenheimer Feld 460, 69120 Heidelberg, Germany

**Keywords:** Exosomes, Intercellular communication, Proteomics, Transforming Growth Factor Beta Receptor Type 2, DNA mismatch repair deficiency, Microsatellite instability, Colorectal cancer

## Abstract

**Background:**

Colorectal cancers (CRCs) that lack DNA mismatch repair function exhibit the microsatellite unstable (MSI) phenotype and are characterized by the accumulation of frameshift mutations at short repetitive DNA sequences (microsatellites). These tumors recurrently show inactivating frameshift mutations in the tumor suppressor Transforming Growth Factor Beta Receptor Type 2 (TGFBR2) thereby abrogating downstream signaling. How altered TGFBR2 signaling affects exosome-mediated communication between MSI tumor cells and their environment has not been resolved. Here, we report on molecular alterations of exosomes shed by MSI cells and the biological response evoked in recipient cells.

**Methods:**

Exosomes were isolated and characterized by electron microscopy, nanoparticle tracking, and western blot analysis. TGFBR2-dependent effects on the cargo and functions of exosomes were studied in a MSI CRC model cell line enabling reconstituted and inducible TGFBR2 expression and signaling. Microsatellite frameshift mutations in exosomal and cellular DNA were examined by PCR-based DNA fragment analysis and exosomal protein profiles were identified by mass spectrometry. Uptake of fluorescent-labeled exosomes by hepatoma recipient cells was monitored by confocal microscopy. TGFBR2-dependent exosomal effects on secreted cytokine levels of recipient cells were analyzed by Luminex technology and ELISA.

**Results:**

Frameshift mutation patterns in microsatellite stretches of *TGFBR2* and other MSI target genes were found to be reflected in the cargo of MSI CRC-derived exosomes. At the proteome level, reconstituted TGFBR2 expression and signaling uncovered two protein subsets exclusively occurring in exosomes derived from TGFBR2-deficient (14 proteins) or TGFBR2-proficient (five proteins) MSI donor cells. Uptake of these exosomes by recipient cells caused increased secretion (2–6 fold) of specific cytokines (Interleukin-4, Stem Cell Factor, Platelet-derived Growth Factor-B), depending on the TGFBR2 expression status of the tumor cell.

**Conclusion:**

Our results indicate that the coding MSI phenotype of DNA mismatch repair-deficient CRC cells is maintained in their exosomal DNA. Moreover, we uncovered that a recurrent MSI tumor driver mutation like TGFBR2 can reprogram the protein content of MSI cell-derived exosomes and in turn modulate the cytokine secretion profile of recipient cells. Apart from its diagnostic potential, these TGFBR2-dependent exosomal molecular and proteomic signatures might help to understand the signaling routes used by MSI tumors.

**Graphical Abstract:**

Fricke et al. uncovered coding microsatellite instability-associated mutations of colorectal tumor driver genes like TGFBR2 in MSI tumor cellderived exosomes. Depending on the TGFBR2 expression status of their donor cells, shed exosomes show distinct proteomic signatures and promote altered cytokine secretion profiles in recipient cells.
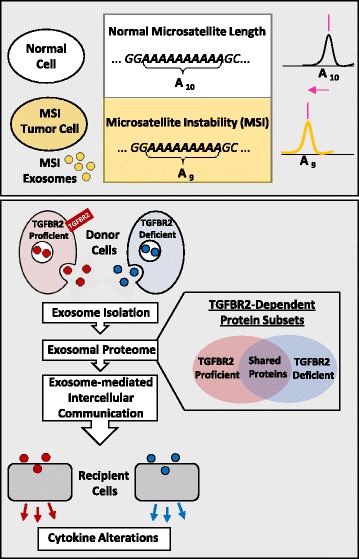

**Electronic supplementary material:**

The online version of this article (doi:10.1186/s12964-017-0169-y) contains supplementary material, which is available to authorized users.

## Plain English summary

A subset of colorectal cancers show the so-called microsatellite instability (MSI) phenotype that is characterized by the accumulation of tumor cell-specific genetic alterations at repetitive DNA sequences. Like many other normal and malignant cells, these MSI tumor cells release 30–150 nm sized vesicles, termed exosomes, to communicate with other cells locally and at distant sites. How MSI tumor cell-specific genetic alterations in the parental cells impact the biology and function of shedded exosomes and in turn affect responder cells remains unresolved.

To address this question, we have isolated and characterized exosomes from MSI colorectal cancer cell lines. We uncovered that the MSI phenotype of the parental cells is shared by their secreted exosomes. It also was observed that the expression status of a tumor driver gene in MSI tumor cells led to specific alterations in the protein content of the released exosomes which in turn elicit a biological response in recipient cells by changing their cytokine secretion profile. Hence, the described molecular and proteomic signatures transmitted by MSI tumor cell-specific exosomes provide novel insights into the biological messages sent by their donor cells and might facilitate the development of new diagnostic and therapeutic approaches.

## Background

Adaptation of tumor cells to their continuously changing microenvironment is reflected by genetic and epigenetic alterations and usually accompanied by significant changes in their cellular phenotype. Among several other mechanisms, extracellular vesicle shedding and release of their cargo to target cells can account for these effects [1, 2]. Exosomes constitute a distinct subset of extracellular vesicles about 30–150 nm in diameter and are released by all normal and neoplastic cells. They can accommodate proteins, nucleic acids, lipids, and metabolites [3] and are involved in different biological functions like intercellular communication, coagulation, and immune response modulation. Accumulating evidence suggests that exosomes play a key role in cancer [4]. It has been demonstrated that cancer cell-derived exosomes can transfer oncogenic proteins and nucleic acids to recipient cells, thereby promoting tumor growth, metastasis, and drug resistance [5–7]. For example, exosomes derived from breast cancer cells but not normal cells contain the complete pre-miRNA processing machinery which enables them to alter the transcriptome of target cells in a Dicer-dependent manner, thereby stimulating non-tumorigenic epithelial cells to form tumors [8]. Likewise, colon cancer cell-derived exosomes have been found to be enriched in ΔNp73 mRNA and the proliferation potential of target cells is greatly enhanced by incubation with ΔNp73-containing exosomes [9]. Thus, most of the genetic alterations that exist in the DNA, RNA or protein of the donor cells also occur in the exosomes derived thereof and contribute to the pathogenesis of cancer.

The pathogenesis of DNA mismatch repair-deficient (dMMR) colorectal cancers (CRC) has gained increasing attention, because of distinct clinico-histopathological and molecular features. When compared to their MMR-proficient counterparts they have a more favorable prognosis [10], show an altered chemo-responsiveness [11, 12], have a lower propensity to form distant metastases [13, 14], and exhibit an intense inflammatory response with tumor-infiltrating lymphocytes [15, 16]. As a molecular hallmark, these dMMR tumor cells accumulate numerous somatic small insertion/deletion mutations predominantly at short, repetitive DNA sequences (microsatellites), thereby manifesting the microsatellite instability (MSI) phenotype [17, 18].

When this instability occurs at coding mononucleotide repeats (cMNR), affected genes will generate transcripts with premature termination codons (PTC) that are usually recognized and degraded by the cellular nonsense-mediated RNA decay system [19, 20], but upon translation can give rise of truncated proteins with highly immunogenic frameshift peptide tails [21]. Several studies have identified a large number of cMNR harboring genes frequently affected by frameshift mutations in MSI colorectal tumors [22, 23], but only a limited set of recurrently mutated genes are considered to drive MSI tumorigenesis.

One of these prime MSI targets is the gene encoding the Transforming Growth Factor Beta Receptor Type 2 (TGFBR2), which is part of a key signaling pathway in colon epithelial cells. In canonical TGFBR2-mediated signaling, binding of the TGF-ß1 ligand to this transmembrane receptor causes hetero-tetramerization with type 1 (TGFBR1) receptors, that upon phosphorylation triggers SMAD-mediated signal propagation and execution of cell context-dependent expression programs [24, 25]. Altered TGFBR2 signaling in tumor cells can modulate a wide range of processes like epithelial-to-mesenchymal transition (EMT), migration and invasion, angiogenesis, immunomodulation, and cytokine secretion. Biallelic cMNR frameshift mutations within the *TGFBR2* gene arise recurrently in most MSI colorectal tumors and are considered to drive MSI tumorigenesis [26]. In the present study, we explored whether the cellular MSI phenotype is maintained in exosomes and how MSI driver mutations in a major signaling pathway, as exemplified by the TGFBR2 tumor suppressor, can alter the exosomal content of MSI tumor cells and in turn elicit a biological response in specific target cells. It turned out, that the MSI status and the cMNR frameshift mutation allele patterns of MSI colorectal cancer cells is reflected by their shed exosomes. Moreover, using our MSI colorectal cancer cell line model system (HCT116-TGFBR2) that enables the analysis of TGFBR2-dependent cellular alterations in an isogenic background [27] we uncovered distinct differences in exosomal protein signatures depending on the TGFBR2 expression status of their donor cells. Similarly, these exosomes cause significant alterations in the cytokine secretion profile of HepG2 recipient cells in a TGFBR2-dependent manner with PDGF-B exhibiting the most prominent increase in protein expression levels. These results provide strong evidence for TGFBR2 being a potent modulator of exosomal protein content and a modulator of cytokine response in specific target cells.

## Methods

### Cell culture

dMMR CRC cell lines (HCT116, RKO, LoVo) and the MMR-proficient CRC cell line (SW948) were obtained from ATCC. The generation of the doxycycline-inducible cell line model system HCT116-TGFBR2 was reported previously [27]. KM12 cells were kindly provided by I.J. Fidler and HepG2 by K. Breuhahn. Cells were grown in RPMI 1640 (LoVo, KM12, RKO, HepG2) or DMEM (HCT116, HCT116-TGFBR2) medium supplemented with 10% FBS, 100 U/ml penicillin and 100 μg/ml streptomycin (Thermo Fisher Scientific Inc., USA) using standard conditions.

### Isolation of exosomes

dMMR CRC cell lines were plated on T175 flasks and grown in complete medium as described above until they reached approximately 80–90% confluency. Cells were washed twice with phosphate-buffered saline (PBS) and cultured for 16 h in minimal volumes (17 ml/T175 flask) of serum-free medium. To investigate TGFBR2-dependent exosomal alterations, HCT116-TGFBR2 cells were cultured in the presence of TGF-ß1 (10 ng/ml) with or without doxycycline (Dox, 0.5 μg/ml). Cell culture media were collected and subjected to sequential centrifugations to remove floating cells (480 × g, 4 °C, 10 min) and cellular debris (2000 × g, 4 °C, 10 min). Supernatants were then passed through a 0.22 μm filter to reduce microparticle contamination and filtrates were concentrated to a final volume of 1 ml by 10,000 molecular weight-cutoff Vivaspin 20 centrifugal concentrators (4000 × g, 4 °C, 30 min; Sartorius, Germany). After addition of 500 μl Total Exosome Isolation Reagent (Thermo Fisher Scientific Inc., USA), samples were incubated overnight at 4 °C on a rotating wheel and then centrifuged at 10,000 x g and 4 °C for 1 h. Exosomal pellets were resuspended either (i) in 100 μl PBS for transmission electron microscopy, (ii) in 200 μl PBS for DNA isolation and fragment analysis, (iii) in 100 μl PBS containing 0.5% bovine serum albumin (BSA) for CFSE-labeling/uptake experiments, or (iv) in 60 μl RIPA buffer in the presence of protease inhibitors for Western blot and mass spectrometry analyses. Isolated extracellular vesicles were stored at −80 °C.

### Transmission electron microscopy

Drops of thawed exosome suspensions were left to settle on 100 mesh formvar-coated copper grids (Plano GmbH, Germany), contrasted with 3% aqueous uranyl acetate (negative stain), air dried and visualized using a JEM-1400 transmission microscope (JEOL GmbH, USA) at 80 KV, equipped with a Tietz 2 K digital camera (TVIPS, Germany).

### Western blotting

Western blot analysis was performed as described previously [27]. Briefly, 30 μg protein was separated on 4–12% SDS-PAGE gels (Thermo Fisher Scientific Inc., USA) and electro-blotted onto a nitrocellulose membrane. The following primary antibodies were used: mouse monoclonal CD63 antibody (1:500, 4 °C, overnight; Abcam, UK), mouse monoclonal CD9 antibody (1:200, 4 °C, overnight; Santa Cruz, Germany), and mouse anti-ß-Actin (1:1000, RT, 30 min; MP Biomedicals, USA). Subsequently, blots were incubated for 1 h at RT with a sheep anti-mouse-IgG HRP secondary antibody (1:5000; GE-Healthcare, UK). Signals were detected using Western Lightning Plus ECL (Perkin Elmer, USA).

### Nanoparticle tracking analysis

Size profiling of isolated vesicles was performed in a dilution of 1:5000 by nanoparticle tracking analysis (NTA) using Nanosight LM10 equipped with a 405 nm laser (Malvern Instruments, UK). The analysis was conducted according to the manufacturer’s instructions by analyzing 30 s-measurements with a slider shutter of 1000 at five different positions per sample.

### PCR-based DNA fragment analysis

Total cellular and exosomal DNA was isolated using QIAamp DNA Mini Kit (Qiagen, Germany). After precipitation by ethanol, the cellular and exosomal DNA was used for PCR-based frameshift analysis. PCR amplification of cMNR sequences was performed using specific primers (Additional file 1) designed with primer3 software (http://primer3.ut.ee/) and the following cycler program: 95 °C for 10 min, followed by 40 cycles of 95 °C for 15 s and 60 °C for 1 min and final extension for 7 min at 72 °C. Fragment analysis was carried out on an ABI 3130xl Genetic Analyzer (Applied Biosystems, Germany) using the Genescan Analysis Software (Applied Biosystems, Germany).

### Mass spectrometry

#### Sample preparation

Total protein RIPA lysates of exosomes were precipitated using a methanol-chloroform-water mixture [28] followed by in-solution tryptic digestion. Precipitated exosomal proteins were redissolved in 10 μl 40 mM NH_4_HCO_3_ and treated with 2 μl 10 mM dithiothreitol (DTT) solution in 40 mM NH_4_HCO_3_ at 45 °C for 1 h to completely reduce disulfide bonds. Afterwards, the thiol groups were alkylated by addition of 1 μl 55 mM iodoacetamide solution in 40 mM NH_4_HCO_3_ and 30 min incubation in the dark at 25 °C. After adding 2.5 μl DTT solution, the mixture was incubated for 15 min at 37 °C to let all iodoacetamide react with a thiol group. Digestion was performed with 100 ng trypsin (Promega, USA) in 40 mM NH_4_HCO_3_ solution overnight at 37 °C. To stop the tryptic digestion, 7.5 μl of 0.1% TFA were added to the sample. In total, 1/5 (i.e. 5 μl) of the sample was subjected to the nano-Liquid Chromatography (LC) electrospray ionization MS/MS analysis. For the analysis, four biological replicates of each sample (JK2358_1–4: exosomes derived from dTGFBR2 cells; JK2358_5–8: exosomes derived from pTGFBR2) have been prepared.

#### Electrospray ionization MS/MS analysis

Tryptic peptide mixtures were separated by a nanoAcquity ultra-high-performance LC system. Peptides were trapped on a nanoAcquity C18 column (180 μm × 20 mm, 5-μm particle size). The liquid chromatography separation was performed on a C18 column (BEH 130 C18, 100 μm × 100 mm, 1.7-μm particle size) with a flow rate of 400 nl/min. For all samples, the chromatography was carried out using a 3 h gradient of solvent A (98.9% water, 1% acetonitrile, 0.1% formic acid) and solvent B (99.9% acetonitrile and 0.1% formic acid) in the sequence: from 0 to 4% B in 1 min, from 4 to 30% B in 140 min, from 30 to 45% B in 15 min, from 45 to 90% B in 5 min, 10 min at 90% B, from 90 to 0% B in 0.1 min, and 9.9 min at 0% B. The ultra-high-performance LC system was connected online to an LTQ Orbitrap XL mass spectrometer (Thermo Scientific, Germany). The mass spectrometer was operated in the sensitive mode with the adjusted parameters: capillary voltage 2400 V; capillary temperature 200 °C; normalized collision energy 35 V; activation time 30,000 ms. Data were obtained in scan cycles of one Fourier transform MS scan with a resolution of 60,000 at m/z 400 and a range from 370 to 2000 m/z in parallel with six MS/MS scans in the ion trap of the most frequent precursor ions.

#### Database search and evaluation

The MS files generated by Xcalibur software (version 2.0.6) were used for database searches with the MASCOT search engine (version 2.4; Matrix Science) against the Swiss-Prot database (SwissProt version 2013_02 (539165 sequences; 191456931 residues)) [29]. The taxonomy was set to “human”. The peptide mass tolerance was set to 5 ppm and the fragment mass tolerance was adjusted at 0.4 Dalton. Carbamidomethylation of cystein (C) was set as a fixed modification. Variable modifications included oxidation of methionine (M) and deamidation of asparagine (N) and glutamine (Q). One missed cleavage site in the case of partial trypsin hydrolysis was accepted. The false discovery rates (FDRs) at the protein and peptide level were set to 1%. Proteins were considered as identified, if more than one unique peptide had an individual ion score exceeding the MASCOT identity threshold (ion score cut-off 20). Identification under the performed search parameters refers to a match probability of *p* < 0.01, where p is the probability that the observed match is a random event. Candidate proteins were classified as differentially expressed (exclusively in exosomes derived from dTGFBR2 or pTGFBR2 cells), if detected in at least three of four biological replicates. The raw data of MS proteomics are deposited to the ProteomeXchange Consortium via the PRIDE partner repository [30] with the project accession number: PXD005620 and project DOI: 10.6019/PXD005620 [31].

### Fluorescent labeling of isolated exosomes

Exosomes were isolated from HCT116-TGFBR2 cells as described above. To label intra-exosomal proteins, isolated exosomes were incubated in 5 μM 5(6)-carboxyfluorescein diacetate *N*-succinimidyl ester (CFSE; Sigma-Aldrich, Germany) at 37 °C for 30 min in the dark. The reaction was stopped by adding two volumes of RPMI medium. After ultracentrifugation (120,000 × g, 4 °C, 2 h; rotor: 100.2; UZ: TLA-100.2, Beckman Coulter, Germany), exosomes were resuspended in 50 μl cold PBS and used directly for uptake experiments.

### Tracking of exosomes uptake by confocal microscopy

HepG2 cells were seeded in 0.5 ml of exosome-depleted medium onto a glass bottom dish (35 × 10 mm; Greiner Bio-One International GmbH, Austria) and treated with 50 μl of CFSE-labeled exosomes overnight in the dark at 37 °C in 5% CO_2_-atm. Next day, the medium was discarded and the cells were washed with 1 ml PBS. Uptake of exosomes by HepG2 cells was analyzed using a Zeiss LSM 710 ConfoCor3 confocal microscope (Carl Zeiss, Germany) equipped with an argon laser and a Plan-Apochromat 63x/1.40 Oil DIC objective (Carl Zeiss, Germany). Excitation was performed at 488 nm and detection was executed in a filter range of 493 to 630 nm. Images were analyzed using IMAGE J-Fiji software [32].

### Luminex-based cytokine profiling of HepG2 cells

HepG2 cells (8.5 × 10^4^ cells/cm^2^) were cultured in 6-well plates for 24 h. Cells were washed with PBS and grown in exosome-depleted 1% FBS-containing RPMI medium in the presence of HCT116-TGFBR2-derived exosomes (20 μg/ml of exosomal protein determined by Bradford Red assay). After 24 h of exosomal exposure, cell culture supernatants were collected and centrifuged (1000 × g, 4 °C, 15 min) and cell numbers in each well were counted. Supernatants were analyzed for 50 secreted cytokines and chemokines using pre-designed Bio-Plex panels (Bio-Plex Pro Human Cytokine 21-, 27-Plex Panel and ICAM-1-, VCAM-1 Assay; Bio-Rad Laboratories GmbH, Germany) according to the manufacturer’s protocol. The standard curve was prepared as a System; Bio-Rad Laboratories GmbH, Germany) with a 532 nm reporter laser and 635 nm classification laser. Cytokine concentrations [pg/ml] were calculated with Bio-Plex Manager software (version 4.0; Bio-Rad Laboratories GmbH, Germany) by optimizing the standard curves for each cytokine. Cytokine concentrations were normalized to corresponding cell numbers and fold changes (pTGFBR2/dTGFBR2) calculated from normalized values.

### PDGF-B enzyme-linked immunosorbent assay (ELISA)

HepG2 cells were seeded as biological duplicates in 12-well plates at densities of 8.5 × 10^4^ cells/cm^2^. Cells were grown in the presence of exosomes as described for the cytokine profiling. Supernatants were collected, centrifuged (1000 × g, 4 °C, 15 min) and the cell numbers of each well were counted. Supernatants were analyzed using the human PDGF-B ELISA (BlueGene Biotech CO., China) according to the manufacturer’s protocol. The OD of each sample was normalized to the corresponding cell number. After subtracting blank value (medium without cells), log_10_OD values were calculated. For analyzing the concentration [pg/ml] of each sample in accordance with the standard curve, the log_10_OD-values of the biological replicates were averaged arithmetically. Finally, fold changes were determined by calculating a concentration ratio (pTGFBR2/dTGFBR2).

## Results

### Characterization of extracellular vesicles Isolated from dMMR colorectal cancer cell lines

**Fig. 1:**
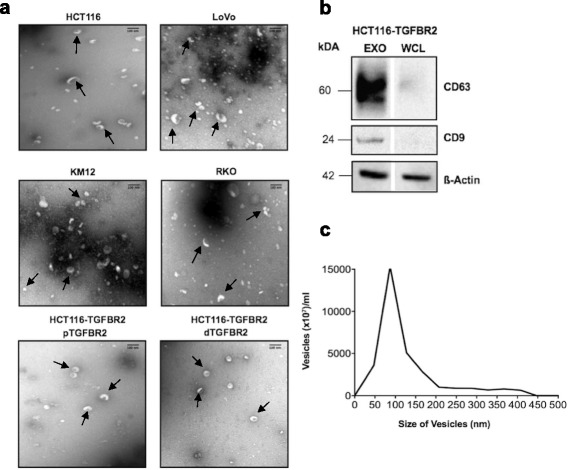
Characterization of isolated exosomes. **a** Transmission electron microscopy (TEM) illustrates the size and shape of exosomes (*indicated by arrows*) isolated from different MSI colorectal cancer cell lines (HCT116, LoVo, KM12, RKO) and from the model cell line HCT116-TGFBR2 (pTGFBR2: TGFBR2-proficient, dTGFBR2: TGFBR2-deficient). Smaller particles represent vesicle fragments resulting from the isolation procedure. Scale bar = 100 nm. **b** Western blot analysis shows tetraspanin (CD63, CD9) marker expression for whole cell lysates (*WCL*) of HCT116-TGFBR2 donor cells and lysates of derived exosomes (*EXO*). ß-actin served as a loading control. **c** Nanoparticle tracking analysis (NTA) indicates size distribution for vesicles isolated from HCT116-TGFBR2 cells (representative of six independent experiments)

To determine the identity of isolated nanovesicles, three different approaches were pursued. First, transmission electron microscopy (TEM) was used for examining the structure of the vesicles. As indicated in Fig. 1a, representative exosomes consisted of cup-shaped closed vesicles that ranged in diameter from 30 to 120 nm and lacked apoptotic bodies and cellular debris. The exosomal appearance was observed in preparations from all dMMR cell lines (HCT116, LoVo, KM12, RKO, HCT116-TGFBR2). Second, Western blot analysis revealed expression of the exosomal marker proteins CD63 and CD9 in protein lysates of exosomes but not in whole cell lysates of HCT116-TGFBR2 donor cells (Fig. 1b). Third, nanoparticle tracking analysis (NTA) confirmed the size distribution of isolated nanovesicles. As shown in Fig. 1c, the major proportion of isolated vesicles ranged in a mono-peak fashion from 50 to 150 nm in diameter. These results indicate the isolation of *bona fide* exosomes from different dMMR donor cells and these exosomes were used for further investigations.

### Exosomes from different dMMR CRC cell lines accumulate frameshift mutations in coding mononucleotide repeats

**Fig. 2:**
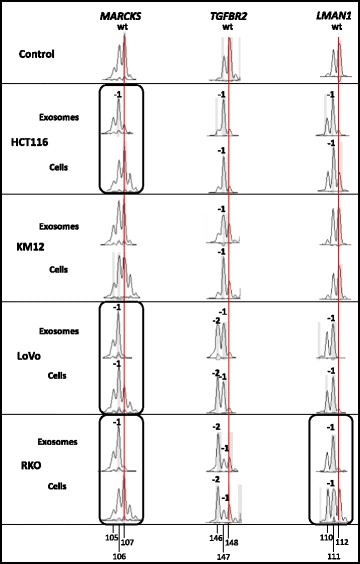
Gene-specific cMNR frameshift mutations in exosomal and cellular DNA of different MSI CRC cell lines. Frameshift mutations are recognized as shifts in allele length as determined by DNA fragment analysis. In the reference cMNR peak pattern of the microsatellite stable cell line SW948 (*control*) the highest peak refers to the normal wildtype (*wt*) allele length (*red vertical line; reference mark*), whereas additional peaks represent PCR-associated artefact peaks. Allele length shifts *(-1, -2*) were scored if novel peaks were obtained in cell lines compared to the microsatellite stable control SW948 cell line, or if the ratio of peak areas of corresponding peaks in cell lines and normal control revealed values ≤0.5 or ≥2. Differences between exosomal and cellular mutant allele pattern (*circled*) are indicated. Allele sizes are given by numbers

In dMMR cells, numerous cMNR-harboring genes are affected by insertion/deletion mutations that constitute the MSI phenotype. Whether genomic DNA harboring such frameshift mutations are encased and detectable in exosomes secreted by dMMR cells is unresolved. Therefore, we isolated exosomal as well as cellular DNA from four dMMR CRC cell lines with known cMNR mutation status (Additional file 2) and examined the cMNR frameshift mutation pattern of three representative MSI target genes (*TGFBR2* [A_10_], *LMAN1* [A_9_], *MARCKS* [A_11_]). The allele profile of the CRC cell line SW948 served as control because these MMR-proficient (pMMR) and microsatellite stable cells lack cMNR mutations (Fig. 2). Using PCR-based DNA fragment analysis, allelic length shifts (-1/-2) affecting three representative cMNR gene sequences (*MARCKS*, *TGFBR2*, *LMAN1*) were observed in exosomes of four different dMMR cell lines. In terms of *TGFBR2* and *LMAN1*, we found almost identical mutant and/or wildtype alleles in MSI cell lines and their derived exosomes. However, specific differences between the exosomal and cellular mutant allele pattern became apparent and are highlighted by circled areas in Fig. 2. For example, only mutant alleles (-1 deletions) affecting the A_11_-cMNR of the *MARCKS* gene were detected in the exosomes of 3/4 cell lines (HCT116, LoVo, RKO), whereas cellular DNA showed either wildtype alleles (HCT116, KM12, RKO) or a mixed normal and mutant allele pattern (LoVo). Similarly, exosomes of 3/4 cell lines (HCT116, LoVo, RKO) only contained mutant (-1) *LMAN1* alleles, while identical mutant or wildtype *LMAN1* allele pattern were observed among exosomal and cellular DNA in 3/4 cell lines (HCT116, KM12, LoVo). Interestingly, the most consistent exosomal and cellular cMNR pattern was identified for the *TGFBR2* gene. All dMMR cell lines exclusively exhibited *TGFBR2* frameshift mutations (−1, −2) and each cell line maintained identical allele pattern intracellularly and within exosomes. These data indicate that the genomic MSI phenotype of dMMR CRC cell lines principally is preserved in the exosomes released by these cells.

### Expression status of the MSI target gene *TGFBR2* modulates the exosomal proteome of dMMR HCT116 CRC cells

Since *TGFBR2* cMNR frameshift mutations are considered drivers of dMMR tumors and HCT116 cells and derived exosomes both express only frameshift-mutated and no wildtype *TGBFR2* gene pattern, we investigated whether the TGFBR2 expression status might affect the exosomal proteome composition of HCT116 cells. To address this question, we used our previously established model system of HCT116-TGFBR2 cells that have been genetically modified to regulate TGFBR2 expression and signaling in a doxycycline-dependent manner [27]. Exosomes were isolated from the supernatant of HCT116-TGFBR2 cells grown in the absence (dTGFBR2) or presence (pTGFBR2) of doxycycline and total exosomal protein lysates were analyzed by mass spectrometry. In total, 1453 different exosomal proteins have been identified. Among these, 1089 overlapping proteins were detected in exosomes derived from both pTGFBR2 and dTGFBR2 HCT116 cells and hence were considered unaffected by TGFBR2 expression of the dMMR donor cell. This shared exosomal proteome encompassed the top 25 exosomal cargo proteins that often have been identified in previous studies (www.exocarta.org). Furthermore, classical exosomal marker proteins like CD63, CD81, and TSG101 have been detected under both conditions thereby confirming the validity of our experimental approach.

**Fig. 3:**
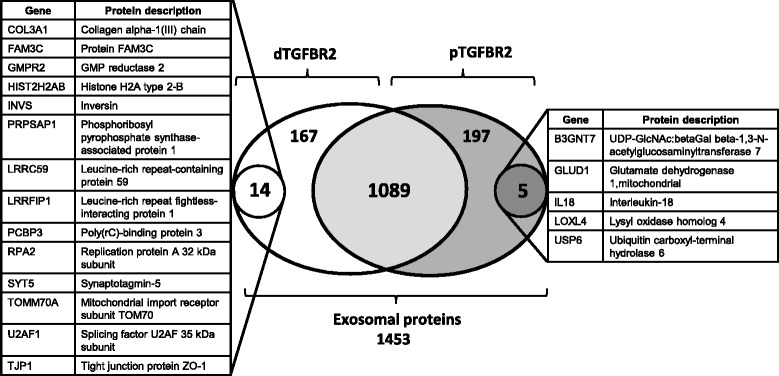
TGFBR2-dependent exosomal proteome profile. Numbers refer to exosomal proteins that comprise the total proteome (*n* = 1453) or define distinct protein subsets, whose expression is either shared by (*n* = 1089) or restricted to exosomes released by TGFBR2-deficient (*n* = 167) or TGFBR2-proficient (*n* = 197) donor cells. From these two latter proteomes two highly specific protein subsets emerged based on more stringent expression criteria (expression in at least three of four biological replicates, see Methods). Individual protein descriptions and gene names are listed

In contrast to this shared proteome, a subset of 364 exosomal proteins was found to be associated with the TGFBR2 expression status of HCT116-TGFBR2 donor cells. In particular, 167 exosomal proteins were identified to be exclusively expressed in exosomes derived from dTGFBR2 cells, whereas 197 proteins remained restricted to exosomal vesicles shed by pTGFBR2 donor cells (Fig. 3). Applying further specific selection criteria revealed a small subset of exosomal proteins (*n* = 19) that presented consistent (3/4 biological replicates) TGFBR2-dependency and hence marked the exclusive TGFBR2-dependent exosomal proteome (Fig. 3, Table 1). The majority of these proteins were found to be expressed solely in dTGFBR2 exosomes (14/19) and thus might have potential impact on the pathogenesis of most MSI tumors that lack normal TGFBR2 function. Identified proteins include factors involved in RNA binding/processing, and transcriptional regulation (U2AF1, PCBP3), migration, extracellular matrix remodeling (COL3A1), epithelial-to-mesenchymal transition (FAMC3), chromatin structure (HIST2H2AB), cell junction structure (TJP1), regulators of cytoskeletal organization (LRRFIP1, INVS), nuclear/mitochondrial import (LRRC59, TOMM70A), DNA replication (RPA2), and vesicular trafficking (SYT5) as well as enzymes for purine nucleotide synthesis (GMPR2, PRPSAP1).

**Table 1:** TGFBR2-dependent exosomal proteome of HCT116 MSI tumor cells

Uni_Prot	Gene	Protein description	Scores	Mass [Da]	Sign Prot matches	Sign Prot sequences	Coverage [%]
dTGFBR2							
CO3A1_HUMAN	COL3A1	Collagen alpha-1(III) chain	34 47 42	139733	1 0 1	1 0 1	0.8 1.4 0.8
FAM3C_HUMAN	FAM3C	Protein FAM3C	47 33 52	24950	1 1 1	1 1 1	5.7 5.7 5.7
GMPR2_HUMAN	GMPR2	GMP reductase 2	49 37 53	38363	0 1 0	0 1 0	8.3 3.4 8.3
H2A2B_HUMAN	HIST2H2AB	Histone H2A type 2-B	245 261 293 291	13987	8 7 8 9	4 4 4 4	52.3 52.3 52.3 52.3
INVS_HUMAN	INVS	Inversin	31 36 42	118837	0 2 2	0 1 1	0.6 0.6 0.6
KPRA_HUMAN	PRPSAP1	Phosphoribosyl pyrophosphate synthase-associated protein 1	39 60 64	39654	1 1 1	1 1 1	6.5 10.4 6.5
LRC59_HUMAN	LRRC59	Leucine-rich repeat-containing protein 59	32 52 79	35308	1 1 1	1 1 1	3.3 7.2 9.1
LRRF1_HUMAN	LRRFIP1	Leucine-rich repeat flightless-interacting protein 1	55 37 75	89826	1 1 1	1 1 1	1.5 1.5 3.1
PCBP3_HUMAN	PCBP3	Poly(rC)-binding protein 3	209 232 212	39725	5 7 7	3 4 4	13.7 17.5 15.4
RFA2_HUMAN	RPA2	Replication protein A 32 kDa subunit	58 45 94	29342	1 1 2	1 1 2	4.4 4.4 12.6
SYT5_HUMAN	SYT5	Synaptotagmin-5	50 54 43	43216	1 1 1	1 1 1	1.6 1.6 1.6
TOM70_HUMAN	TOMM70A	Mitochondrial import receptor subunit TOM70	48 83 53	68096	1 1 1	1 1 1	3 3 3
U2AF1_HUMAN	U2AF1	Splicing factor U2AF 35 kDa subunit	40 35 51	28368	0 1 1	0 1 1	11.2 3.8 11.2
ZO1_HUMAN	TJP1	Tight junction protein ZO-1	40 61 83	195682	1 1 1	1 1 1	0.7 1.4 1.4
pTGFBR2							
B3GN7_HUMAN	B3GNT7	UDP-GlcNAc:betaGal beta-1,3-N-acetylglucosaminyltransferase 7	30 44 57	46471	0 1 1	0 1 1	4.7 4.7 4.7
DHE3_HUMAN	GLUD1	Glutamate dehydrogenase 1, mitochondrial	91 60 49	61701	2 1 0	2 1 0	5.9 3.4 3.9
IL18_HUMAN	IL-18	Interleukin-18	42 44 36	22597	1 1 1	1 1 1	13.5 13.5 13.5
LOXL4_HUMAN	LOXL4	Lysyl oxidase homolog 4	110 221 169	86425	2 3 3	2 3 3	4.8 9.1 10.3
UBP6_HUMAN	USP6	Ubiquitin carboxyl-terminal hydrolase 6	34 34 31	160781	1 2 1	1 1 1	0.4 0.4 0.4

Different numbers in each column (scores, sign. prot. matches, sign. prot. sequences, coverage) refer to values for three or four biological replicates

In contrast, a much smaller number of exosomal proteins (5/19) were detected exclusively in vesicles secreted by pTGFBR2 HCT116 cells. Among these proteins were the pro-inflammatory cytokine interleukin 18 (IL-18) as well as four different enzymes like the beta 1,3-N-acetylglucosaminyltransferase 7 (B3GNT7), the mitochondrial glutamate dehydrogenase 1 (GLUD1), the lysyl oxidase-like 4 protein (LOXL4), and the ubiquitin-specific protease 6 (USP6). These observations suggest that the TGFBR2 expression status of MSI tumor cells determines the expression of specific protein subsets in derived exosomes that are expected to elicit TGFBR2-dependent responses in recipient cells.

### Exosomes of MSI tumor cells cause alterations in the cytokine profile of HepG2 target cells

**Fig. 4:**
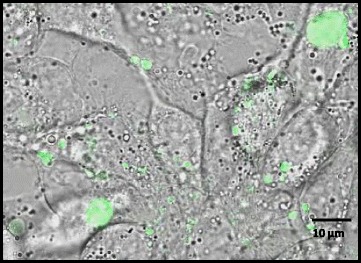
Uptake of exosomes by HepG2 recipient cells. HCT116-TGFBR2 derived exosomes were labeled with CFSE (green-fluorescent dye) and their uptake by HepG2 recipient cells was monitored by confocal microscopy. Scale bar = 10 μm


Since mutation in the TGF-ß pathway are among the most frequent genetic alterations found in CRCs and these tumors frequently metastasize to the liver, we examined the ability of HCT116-TGFBR2-derived exosomes to induce a biological response in HepG2 recipient cells. First, we analyzed the uptake of exosomes by these target cells using CFSE-labeled exosomes derived from HCT116-TGFBR2 cells. CFSE is a membrane permeable dye, which allows exosomal esterases to hydrolyze the dye by removing diacetate residues intra-exosomally. This reaction activates the green fluorescence of CFSE that is coupled to the amino ends of exosomal proteins. The appearance of green-fluorescent exosome-derived proteins inside of HepG2 cells was monitored by confocal microscopy (Fig. 4, Additional file 3).

Additional file 3: Time lapse analysis of CFSE-labeled exosome uptake. Confocal laser scanning microscopy in the 3D-scanning modus was applied to continuously monitor CFSE-labeled exosomes by HepG2 recipient cells. The green fluorescence refers to intracellular signal accumulation of exosomes. Scale bar = 10 μm. (MOV 7290 kb)


These experiments clearly demonstrate the transmission of exosomal cargo proteins to HepG2 cells. This exosomal delivery of bioactive molecules is powerful to modulate various processes in recipient cells. One pronounced effect is the ability of exosomes to modify the spectrum of secreted cytokines upon cargo transmission. For example, it is known that exosomes derived from lung cancer cells can stimulate pro-inflammatory cytokine production in mesenchymal cells and hence favoring a tumor supportive milieu [33]. Since exosomes are known to modulate the spectrum of secreted cytokines in recipient cells, HepG2 cells were exposed to exosomes and any changes in the protein levels of 50 different secreted cytokines and chemokines were determined by Luminex technology.

As shown in Table 2, several cytokines exhibited significantly decreased or increased expression levels (cut off >1.5 fold) in a TGFBR2-dependent manner. Apart from IL-4 (2-fold) and SCF (2.5-fold), the secretion of Platelet-derived Growth Factor-B (PDGF-B) showed the most prominent TGFBR2-dependent response (6-fold) after exosomal exposure. Using a PDGF-B specific ELISA as an alternative method, confirmed the results obtained by Luminex assay. However, the PDGF-B concentration in the supernatant of HepG2 cells exposed to exosomes from dTGFBR2 cells was twice as high in the ELISA assay compared to the Luminex assay (87.61 pg/ml and 40.47 pg/ml, respectively) resulting in a decreased observed ratio of 3-fold (Table 3).

**Table 2:** Luminex assay-based analysis of cytokine and chemokine secretion levels in response to exosomal exposure

	Cytokine/Chemokine concentration [pg/ml]									
	IL-4	IL-9	IL-10	IL-17	G-CSF	IP-10	PDGF-B	TNF-a	MIF	SCF
pTGFBR2	0.92	6.21	47.03	23.98	18.90	6.64	255.93	13.62	589.72	85.33
dTGFBR2	0.46	3.73	31.42	15.25	10.06	3.83	40.47	8.53	333.29	33.80
Fold change (pTGFBR2/dTGFBR2)	2.00	1.66	1.50	1.57	1.88	1.73	6.32	1.60	1.77	2.52

Fold change values indicate the ratio of secreted cytokine levels by HepG2 recipient cells upon exposure to exosomes derived from pTGFBR2 HCT116 cells in comparison to exosomes isolated from dTGFBR2 donor cells. G-SCF: Granulocyte Colony Stimulating Growth Factor; IP-10: Interferon-Gamma-Inducible Protein 10; PDGF-B: Platelet-derived Growth Factor-B; MIF: Macrophage Migration Inhibitory Factor; SCF: Stem Cell Factor

**Table 3:** ELISA-based validation of PDGF-B secretion

PDGF-B concentration [pg/ml]	
pTGFBR2	263.28
dTGFBR2	87.61
Fold change (pTGFBR2/dTGFBR2)	3.01

Validation of PDGF-B secretion by HepG2 cells in response to exosomal treatment in a TGFBR2-dependent manner

In summary, these results demonstrate that the donor cell-dependent TGFBR2 expression status determines the cargo composition of secreted exosomes and that these exosomes are capable to modulate the biological/cytokine local environment of recipient cells.

## Discussion

In this study, we show for the first time that exosomes harbor the same gene-specific cMNR frameshift mutations that have been identified in their cells of origin. One prominent example is the *TGFBR2* gene, a recurrently mutated driver of MSI tumorigenesis. Although detectable at the DNA level, we did not observe any frameshift proteins in the exosomal proteome, which is well in line with previous studies from us and others that failed to detect such truncated proteins in total cell lysates from various dMMR MSI tumor cell lines [34]. The genetic alterations, i.e. coding microsatellite mutations, that we have identified in the exosomes of dMMR tumor cells are MSI tumor-specific and reflect the mutation profile of the donor cells. Preliminary experiments suggest that this also applies to diagnostic microsatellite markers routinely used for MSI classification of tumor specimens (*Fricke* et al., unpublished data). Evidently, this indicates the potential clinical and diagnostic utility of MSI-tumor cell derived exosomes. So far, our findings only apply to established MSI colorectal cancer cell lines and certainly need to be confirmed in exosomes isolated from liquid biopsies of MSI tumor patients. Building up on our findings, exosomes from cultured MSI cell lines might prove useful to define assay thresholds for the detection of cMNR mutations in specific MSI driver genes. Moreover, monitoring of exosomal MSI profiles might facilitate the development of strategies for enrichment of MSI-tumor specific exosomes.

Apart from this MSI-specific molecular fingerprint of exosomes, our results also provide experimental evidence for alterations at the proteome level. This was observed by focusing on the TGFBR2 signal transducer, a frequent mutation target and driver of MSI colorectal tumorigenesis [26, 35]. The results obtained in this study, clearly shows that the TGFBR2 expression/signaling status in the parental cells determined the protein profile of exosomes secreted by these cells. Although the results of our mass spectrometry data revealed no quantitative information, four enzymes (B3GNT7, GLUD1, LOXL4, USP6) and one pro-inflammatory cytokine (IL-18) were exclusively identified in exosomes released from pTGFBR2 cells. The cytokine IL-18 is a mediator of inflammation and an interferon-gamma-inducing factor that is involved in various processes of epithelial repair and collagen production that is emphasized by the prevention of TGF-ß-induced collagen gene expression due to IL-18 activity [36]. Moreover, IL-18 is a mediator of immune responses to eliminate cancer cells effectively [37]. However, IL-18 can also promote tumorigenesis by inducing angiogenesis, migration, metastasis, proliferation, and immune-escape [38, 39].

Apart from IL-18, the enzyme beta 1,3-N-acetylglucosaminyltransferase 7 (B3GNT7) was detected specifically in exosomes derived from pTGFBR2 HCT116-TGFBR2 cells. It has been reported that B3GNT7 is abundantly expressed in normal colon cells and significantly suppressed in colon cancer tissues by epigenetic alterations with effects on the metastatic spread potential [40]. Migratory and metastatic behavior of cancer cells can be further influenced by the lysyl oxidase-like 4 protein (LOXL4), an enzyme that is highly involved in the biogenesis of connective tissue and matrix re-modelling [41] and which has been identified as a protein in TGFBR2 expressing cell-derived exosomes. Another enzyme that is solely expressed in a pTGFBR2 manner is mitochondrial glutamate dehydrogenase 1 (GLUD1). This enzyme is involved in various metabolic processes and associated with poor prognosis in CRC [42]. Aberrant energy metabolism is a hallmark of many cancer cells [43] and plays a role in colorectal tumorigenesis. Recently, it was shown that over-expression of glutamate dehydrogenase (GDH) is associated with CRC metastasis and poor prognosis [44]. Finally, the expression of the enzyme ubiquitin-specific protease 6 (USP6) was exclusively identified in a pTGFBR2 fashion. USP6 can exert different functions by acting as an oncogene, promoting WNT signaling or modulating migration and cytokinesis [45, 46].

A larger subset of 14 proteins has been detected only in exosomes derived from dTGFBR2 cells. This protein subset is of particular clinical interest because it should reflect more accurately the situation existing in most primary MSI colorectal tumors with recurrent loss of TGFBR2 function. The majority of these candidate proteins are involved in migratory processes including EMT. For example, the candidate protein Leucine-rich repeat fightless-interacting protein 1 (LRRFIP1) is known to play a pivotal role in cytoskeletal organization. Using Vesiclepdia and Exocarta Databases [47, 48], this protein has already been identified in the cargo of CRC-derived exosomes [49]. The function of LRRFIP1 in cellular polarity/organisation is exemplified by a recent study that demonstrated LRRFIP1-stimulated CRC metastasis and invasion of hepatocytes through integrin-dependent RhoA activation [50]. Interestingly, RhoA can also be activated by Collagen alpha-1(III) chain (COL3A1) [51], another candidate exclusively identified from the cargo of exosomes derived from dTGFBR2 cells. The COL3A1 protein is upregulated in advanced ovarian carcinoma [52] and a known stimulator of growth acceleration in human osteoblastic cells [53]. The small GTPase RhoA is involved in various cellular processes and a prominent regulatory factor of cytoskeletal dynamics by the induction of stress fibers [54]. Furthermore, RhoA is considered as an essential driver of TGF-ß induced EMT [55].

Further candidates include the FAM3C protein, a promoter of EMT and metastatic progression [56, 57] that has previously been detected in exosomes from other CRC cell lines [49]. Studies have shown that cytoplasmic FAM3C expression might serve as a prognostic factor in colorectal malignancies [58]. Moreover, it has been proposed that elevated concentrations of FAM3C in the secretome of highly autophagic melanoma cell lines could serve as candidate autophagy biomarker [59]. The expression and metastatic proficiency of FAM3C is further regulated by TGF-ß at a post-transcriptional level [57, 60].

Another candidate protein linked to cellular polarity and cell-to-cell contacts is the tight junction protein ZO-1 (TJP1) that is also exclusively expressed in the dTGFBR2 exosomal proteome. Recently, it was shown that TGF-ß increases the expression of TJP1 and enhances cell motility of lung cancer cells [61]. Expression of the Inversin (INVS) protein also was restricted to exosomes derived from dTGFBR2 cells. Concerning the role of INVS in the organization of the cytoskeleton, it has been suggested that INVS modulates cellular polarity through positioning the mitotic spindle and at least partially by transcriptional regulation of genes involved in WNT signaling and pathways associated with the maintenance of the actin cytoskeleton/network and the migratory cellular potential [62, 63]. In another study, the migratory and metastatic potential of breast cancer is also correlated with another candidate protein, Leucine-rich-repeat-containing protein 59 (LRRC59), uncovered specially in the exosomal cargo derived from dTGFBR2 cells [64]. Although not yet confirmed by functional studies, our identified candidates suggest that exosomes derived from dTGFBR2 MSI CRC cells might modulate the migratory potential and/or cytokine profile of target cells.

Target cell changes due to exosomal exposure have been well described. For example, HepG2 cells can serve as target cells for exosomes derived from CRC cells [65]. Moreover, it was shown that exosomes derived from SW480 CRC cells are capable to stimulate the migratory behavior and thus driving cellular alterations of HepG2 cells [66]. When we examined HepG2 target cells that have been exposed to HCT116-TGFBR2 derived exosomes no obvious morphological changes were observed. Instead, our experiments revealed major differences in the cytokine profile in response to exosomal exposure. Depending on the TGFBR2 expression/signaling status of HCT116-TGFBR2 donor cells, exosomes-treated HepG2 target cells exhibited significantly increased secretion of interleukin-4 (2-fold, IL-4), stem cell factor (2.5-fold, SCF) and Platelet-derived Growth Factor-B (6-fold, PDGF-B). Although previous research could not observe any IL-4-specific cytokine concentration in liver tissue or liver metastasis [67], our results indicate that transmission of exosomes derived from HCT116-TGFBR2 CRC cells impact IL-4 secretion of recipient HepG2 cells in a TGFBR2-dependent fashion. For PDGF-B, the biological response was being validated by ELISA assay (3-fold). The observed difference in the ratio between Luminex and ELISA methodology might relate to different assay reagents such as the capture and reporter antibodies [68].

The exact mechanism how TGFBR2 reconstitution in the donor cells elicits exosome-linked PDGF-B release in HepG2 target cells is still unclear. From our previous work, we know that reconstituted TGFBR2 as well as ACVR2 signaling can cause an upregulation of *PDGF-B* at the transcriptional level in the HCT116 cell line model system [69]. Also, preliminary evidence suggests that *PDGF-B* transcripts are more abundant in exosomes of pTGFBR2 compared to dTGFBR2 HCT116 cells. However, it is not demonstrated by our experiments if these transcripts account for the high levels of PDGF-B secretion from HepG2 target cells. Metabolic labeling experiments could resolve whether this is attributable to exosomal cargo protein delivery to or de novo protein synthesis in recipient cells. Furthermore, the results obtained from HepG2 cells need to be validated in primary hepatocytes.

Several studies have shown that PDGF signaling can contribute to hepatocellular and colorectal tumor biology. For example, PDGF plays a fundamental role in the initiation of TGF-ß-mediated hepatocellular EMT [70]. Also, in colorectal tumors, expression of PDGF receptors is associated with a high metastatic spread potential [71] and expression of the PDGF-B receptor might contribute to aggressive phenotypes of colorectal tumors with mesenchymal characteristics and enhanced metastatic properties [72]. Moreover, increased levels of PDGF-B have been detected in the plasma of colorectal tumor patients [73]. Clearly, the in vitro model system used in the present study is limited to uncover the biological consequences associated with increased levels of cytokines and growth factors like PDGF-B and how exosomal cargo might shape local and distal environments for the benefit of primary MSI tumors. However, our data strongly suggest that TGFBR2-dependent reprogramming of exosomal cargo can convey MSI tumor cell-specific biological properties to specific target cells.

## Conclusion

In conclusion, our data demonstrate that the coding MSI phenotype of dMMR cells is maintained in their secreted exosomes and a recurrent MSI driver mutation not only determines the protein content of MSI exosomes but also alters the cytokine profile of HepG2 recipient cells. Hence, MSI exosomes most likely elicit similar alterations in other potential target cells of endothelial, mesenchymal or hematopoietic origin which, altogether, might provide some mechanistic insights into the specific clinic-histopathological features of these MSI malignancies. Finally, the MSI- and/or TGFBR2-dependent proteome of serum exosomes of MSI tumor patients could serve as a novel source of MSI-specific diagnostic markers.

## Additional files

Additional file 1: Primers used for cMNR fragment analysis of exosomal and cellular DNA. (DOCX 46 kb)

Additional file 2: Gene-specific cMNR frameshift mutant (-1/-2/+1) and wildtype (wt) alleles. (DOCX 46 kb)

## Abbreviations

cMNR: Coding mononucleotide repeat
CRC: Colorectal cancer
dMMR: DNA mismatch repair deficiency
dTGFBR2: TGFBR2-deficient
MSI: Microsatellite instability
pTGFBR2: TGFBR2-proficient
