# Supplementary material for: TGFBR2-dependent alterations of exosomal cargo and functions in DNA mismatch repair-deficient HCT116 colorectal cancer cells
Source: Cell Commun Signal. 2017 Apr 4;15:14. doi: 10.1186/s12964-017-0169-y (PMC5379773; doi:10.1186/s12964-017-0169-y)
Supplement: Supplementary file 2 — Primers used for cMNR fragment analysis of exosomal and cellular DNA. (DOCX 46 kb) [file 12964_2017_169_MOESM1_ESM.docx]

**Additional file 1: Primers used for cMNR fragment analysis of exosomal and cellular DNA.**

| **Genes [cMNR]** | **Sense [5'>3‘]** | **Antisense [5'>3‘]** | **T [°C]** | **Amplicon [bp]** |
| --- | --- | --- | --- | --- |
| **TGFBR2** [A_10_] | F-GCTGCTTCTCCAAAGTGCAT | CAGATCTCAGGTCCCACACC | 60 | 149 |
| **MARCKS** [A_11_] | GACTTCTTCGCCCAAGGC | F-GCCGCTCAGCTTGAAAGA | 60 | 109 |
| **LMAN1** [A_9_] | F-CACCCATGTCAGCTTTGCTA | GGAGGAATTTGAGCACTTTCA | 60 | 114 |
