# Supplementary material for: TGFBR2-dependent alterations of exosomal cargo and functions in DNA mismatch repair-deficient HCT116 colorectal cancer cells
Source: Cell Commun Signal. 2017 Apr 4;15:14. doi: 10.1186/s12964-017-0169-y (PMC5379773; doi:10.1186/s12964-017-0169-y)
Supplement: Supplementary file 3 — Gene-specific cMNR frameshift mutant (-1/-2/+1) and wildtype (wt) alleles. (DOCX 46 kb) [file 12964_2017_169_MOESM2_ESM.docx]

**Additional file 2:** **Gene-specific cMNR frameshift mutant (-1/-2/+1) and wildtype (wt) alleles.**

|  | **TGFBR2** [A_10_] | **MARCKS** [A_11_] | **LMAN1** [A_9_] |
| --- | --- | --- | --- |
| **HCT116** | -1/-1 | -1/wt | -1/wt |
| **KM12** | -1/wt | -1/wt | wt/wt |
| **LoVo** | -1/-2 | -1/-1 | -1/-1 |
| **RKO** | -1/-2/wt | wt/wt | -1/wt/+1 |

Gene-specific cMNR frameshift mutant (-1/-2/+1) and wildtype (wt) alleles in genomic DNA of different MSI colorectal cancer cell lines.
